# Analgesia in trauma patients treated by advanced life support nursing units: a retrospective pre-post intervention observational study

**DOI:** 10.1590/1518-8345.7899.4903

**Published:** 2026-08-12

**Authors:** Juan José García-Líndez, Ana María Uréndez-Ruíz, Juan Carlos Prieto-Gálvez, Jaume Ponce-Taylor, Francisco José Cereceda-Sánchez

**Affiliations:** 1 SAMU 061 Baleares, IB-Salut, Palma de Mallorca, Islas Baleares, Spain.; 2 Universidad de las Islas Baleares, Facultad de Enfermería y Fisioterapia, Palma de Mallorca, Islas Baleares, Spain.

**Keywords:** Nursing, Emergency Medical Services, Ambulances, Pain Measurement, Analgesia, Wounds and Injuries

## Abstract

**Objective:**

to assess whether the administration of different groups of intravenous analgesics in advanced life support nursing units to patients with acute trauma and moderate or severe pain significantly reduced perceived pain.

**Method:**

retrospective and analytical observational study, pre-post intervention type. Data were collected from the information system and medical records. Descriptive statistics and nonparametric tests were used to analyse pain levels at beginning and end of care, their association with the drugs administered, and care times.

**Results:**

data from 164 patients were evaluated. The mean age was 54±21.49 years, and 48.78% were women. The initial score on a verbal numerical rating scale was = 8,48±1,04 points, while the final score was 4.09±2.32 points, with a statistically significant difference between both moments (p <0.001). Opioid treatments showed greater analgesic efficacy than non-opioid analgesics (p=0.014).

**Conclusion:**

the administration of analgesia by nurses in advanced life support units in the out-of-hospital setting achieves a reduction in perceived pain in acute trauma. Opioids, alone or in combination with non-opioid analgesics, are associated with greater pain reduction.

## Highlights

**(1)** Intravenous analgesia significantly reduces pain in trauma patients. **(2)** Opioids, alone or in combination with non-opioid analgesics, provide greater pain relief. **(3)** The advanced life support nursing unit is safe and effective in administering analgesia. **(4)** The administration of intravenous analgesia is not associated with adverse events in the short term. **(5)** Prolonged care time correlates with greater reduction in traumatic pain.

## Introduction

In Europe, it is estimated that around 38 million people are treated annually in emergency departments for a traumatic event^1^. Acute pain is a frequent reason for consultation among patients who have suffered trauma. The documented prevalence is around 70% in the prehospital setting, and the literature suggests that it can reach 90%^2^ ^-^ ^5^. In certain European countries, it has been documented that only between 14% and 32% of patients with moderate to severe pain received analgesia during prehospital care^1^. A study conducted in the United States of America (USA) indicated that up to 50% of patients with bone fractures had not received analgesia in emergency departments^6^. In the prehospital care phase of severe trauma, early and adequate relief of acute pain is recommended in order to facilitate patient transport to the hospital, ensure their comfort, and reduce the harmful effects of pain and associated stress^7^. Several studies indicate that the use of a nursing-initiated analgesia protocol, both in outpatient settings and in emergency departments, reduces the time until adequate pain management is achieved, reduces pain intensity, and increases patient satisfaction^6^ ^,^ ^8^ ^-^ ^9^.

In pain assessment, unidimensional scales, such as the Verbal Numerical Rating Scale (VNRS) or the visual analogue scale (VAS), are among the most widely used^10^, due to their ease of use and standardisation^7^. The VNRS is the scale currently used for patients treated by SAMU 061 Baleares.

For more than a decade, advanced life support nursing units (ALS-N) have been legally recognized as advanced emergency care resources in the out-of-hospital setting in Spain. These are nursing-led units equipped with the necessary materials to provide the required care in a manner comparable to an advanced life support unit (ALS) with a physician. ALS-N units operate with support and advice from physicians at the Medical Emergency Coordination Centre (MECC) of SAMU 061 Baleares, through telemedicine. When necessary, they receive pharmacological prescriptions from the MECC. This model of care was implemented in the Spanish health system three decades ago, initially without a specific regulatory framework. Since then, it has evolved in different ways in the various self-governing regions. In our region, there have been units of this type since 2018; there are currently two: one in the city of Palma de Mallorca (I210) and another in the town of Sóller (I212).

These ALS-N units consist of a health emergency technician and a nurse. The nurse must have more than one year of previous experience in the service, accredited training in ANLS, specific training in ANLS provided by the training department of SAMU 061 Baleares and must have completed supervised shifts in an ALS-N unit prior to joining, in accordance with the guidelines of the ANLS working group of the *Sociedad Española de Medicina de Urgencias y Emergencias* (SEMES).

The main objective of the study was to assess whether the administration of different groups of intravenous analgesics in advanced life support nursing units to patients with acute trauma and moderate or severe pain significantly reduced perceived pain. The secondary objectives were: (i) to describe the frequency of adverse events after the administration of analgesia; (ii) to determine whether the different groups of analgesics used had similar efficacy in reducing pain; (iii) to assess whether there were differences in the reduction of perceived pain in relation to assistance and transport times between the two nursing life support units studied.

## Methods

### Type of study and location

Retrospective, analytical, single-centre observational pre-post-intervention study, conducted in the context of out-of-hospital emergencies in the cities of Palma de Mallorca and Sóller, on the island of Mallorca, Balearic Islands, Spain.

### Population and study period

Adult patients treated after trauma between January 2019 and December 2023 by one of the two participating ALS-N units. In cases where medication was required, the nurse performed peripheral venous puncture and requested from a MECC physician, via telemedicine, the intravenous analgesia regimen.

### Study participants and selection criteria

The sample was obtained through non-probabilistic convenience sampling. Initially, all adult patients treated by an ALS-N unit after trauma registered in the information system during the study period were included. The inclusion criteria were: patients over 14 years of age treated with any of the ICD codes related to trauma and with clinical criteria suggestive of fracture; having a VNRS for pain recorded at the beginning of care (initial VNRS) equal to or above 6, and a record upon arrival at the hospital (final VNRS). Exclusion criteria were: cases without a complete verbal numerical pain scale record (initial and final VNRS); cases in which intravenous analgesia was not administered; pediatric patients (under 14 years of age); and patients without a record of assistance time (difference between the time of arrival at the incident and the time of transfer to the destination hospital). 

### Study variables

The independent variables were: sociodemographics (gender and age), analgesia administered, initial numerical pain scale score, care unit code, and length of care. The dependent variables were: numerical pain scale score at the end of care and number of adverse events recorded after analgesia administration.

### Data collection and analysis

The data were collected from the MECC’s SENECA^©^ information system and the SAMU 061 Baleares assistance records and were processed anonymously. The analysis was performed using the Just Another Statistical Program (JASP). Quantitative variables were described using mean (), standard deviation (SD), and median; categorical variables were described using frequencies and percentages. Before performing statistical inferences, the normality of the distribution of quantitative variables was assessed using the Shapiro-Wilk test. As normality was not found, nonparametric tests were used: Wilcoxon test (W), Mann-Whitney U test (U), Friedman test (χ2F), Spearman’s rho coefficient (r_s_), and Kruskal-Wallis test (H). In addition, when significant differences were identified, Dunn’s nonparametric *post hoc* test was applied, with Holm and Bonferroni corrections. Associations between variables were evaluated using hypothesis tests with a significance level of p<0.05. In order to reduce reporting bias, non-significant results (p>0.05) are also presented. To make statistical inferences and given the diversity of treatments administered, these were grouped into four categories according to the family of active ingredients and the combinations used during care: non-opioid analgesics (paracetamol, metamizole, dexketoprofen), opioids (morphine, fentanyl, meperidine), opioids combined with analgesics, opioids combined with benzodiazepines (diazepam, midazolam). 

### Ethical aspects

This study complied with the principles of the Declaration of Helsinki and was approved by the Balearic Islands Health Research Ethics Committee (IB5553/24 PI).

## Results

**Figure 1:**
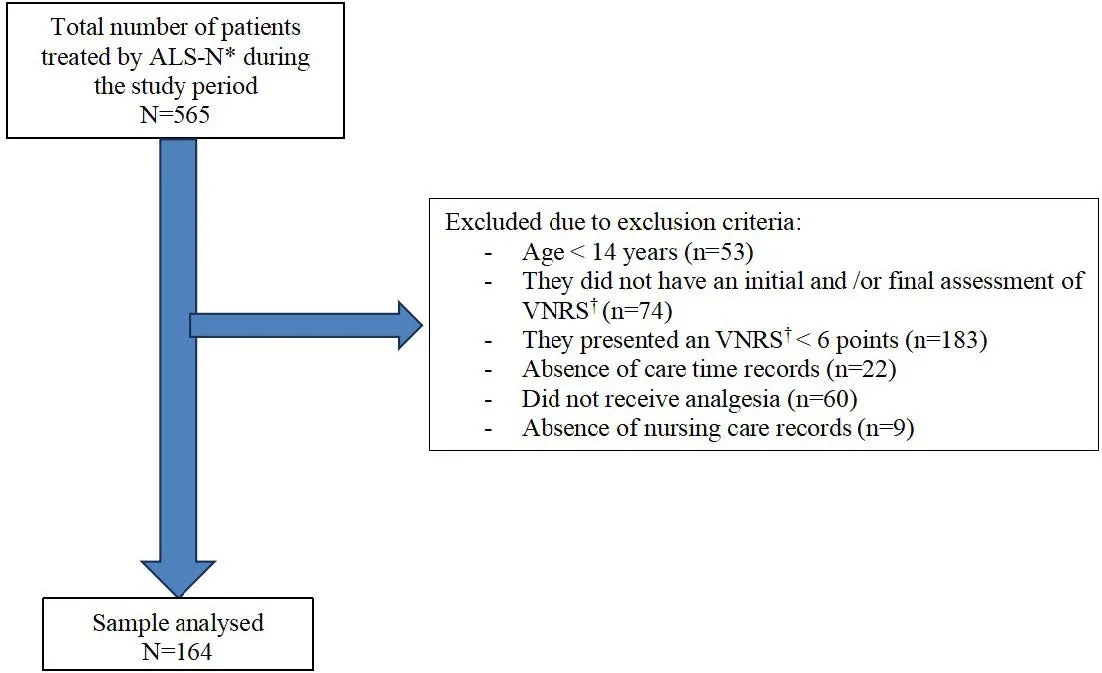
Flowchart of the recruitment of the analysed sample

The total number of patients included in the analysis during the study period is shown in Figure 1.

*ANLS = Advanced Nursing Life Support; ^†^VNS = Verbal Numerical Scale

Table 1 shows the descriptive statistics of the sample and the main variables of the study.

The mean age of patients was 54 ± 21.49 years, and 48.78% were women (n=80). Of the total, 74.39% (n=122) of consultations were performed by unit I210. The initial VNRS score was = 8.48 ± 1.04 points, while the final VNRS score was = 4.09 ± 2.32 points. The reduction after treatment administration was = 4.39 ± 2.39 points. In 5.48% of patients, no reduction in perceived pain was observed (n=9), despite the administration of analgesia.

**Table 1:** Descriptive statistics of the variables analyzed according to advanced life support nursing unit (ALS-N*), with adherence tests (n = 164). Palma de Mallorca, Balearic Islands, Spain, 2024

Variables by resource ALS-N*	Age		Assistance time		VNRS^†^ baseline		VNRS^†^ final		Difference VNRS^†^	
	I210^‡^	I212^§^	I210^‡^	I212^§^	I210^‡^	I212^§^	I210^‡^	I212^§^	I210^‡^	I212^§^
Mode	52.85	65.48	42.58	64.27	9.97	8.0	5.42	2.19	3.79	6.05
Median	52.0	61.5	41.63	64.34	8.0	8.0	4.0	3.0	4.0	6.0
Standard deviation	20.96	22.26	18.81	22.14	1.41	1.41	2.29	2.20	2.35	2.32
Coefficient of variation	0.40	0.37	0.43	0.34	0.16	0.16	0.52	0.67	0.53	0.44
Interquartile range	33.75	35.25	22.44	23.14	2.0	2.0	3.75	3.0	4.0	3.75
Skewness	0.08	-0.15	0.81	0.19	-0.33	-0.38	0.03	0.62	0.29	-0.37
Standard error of skewness	0.21	0.36	0.21	0.36	0.21	0.36	0.21	0.36	0.21	0.36
Kurtosis	-0.94	-1.01	1.4	-0.09	-1.17	-0.98	-0.55	-0.28	-0.18	-0.38
Standard error of kurtosis	0.43	0.71	0.43	0.71	0.43	0.71	0.43	0.71	0.43	0.71
Shapiro-Wilk	0.97	0.96	0.96	0.98	0.84	0.82	0.96	0.92	0.96	0.95
Shapiro-Wilk p-value	0.01	0.22	0.00	0.67	<.001	<.001	0.004	0.008	0.006	0.10
Amplitude	80.0	80.0	104.6	91.4	4.0	4.0	10.0	8.0	11.0	10.0
Minimum	15.0	19.0	8.18	21.38	6.0	6.0	0.00	0.00	-1.00	0.00
Maximum	95.0	99.0	112.8	112.8	10.0	10.0	10.0	8.0	10.0	10.0
First quartile	35.25	43.25	30.16	52.32	8.0	8.0	2.25	2.0	2.0	3.25
Third quartile	69.0	78.5	52.6	75.4	10.0	10.0	6.0	5.0	6.0	7.0

*ALS-N = Advanced Life Support Nursing unit; ^†^VNRS = Verbal Numerical Rating Scale; ^‡^I210 = Advanced life support nursing unit located in the capital, Palma de Mallorca; ^§^I212 = Advanced life support nursing unit located in the town of Sóller

Table 2 shows the frequencies of treatments administered, grouped by family of active ingredients.

**Figure 2:**
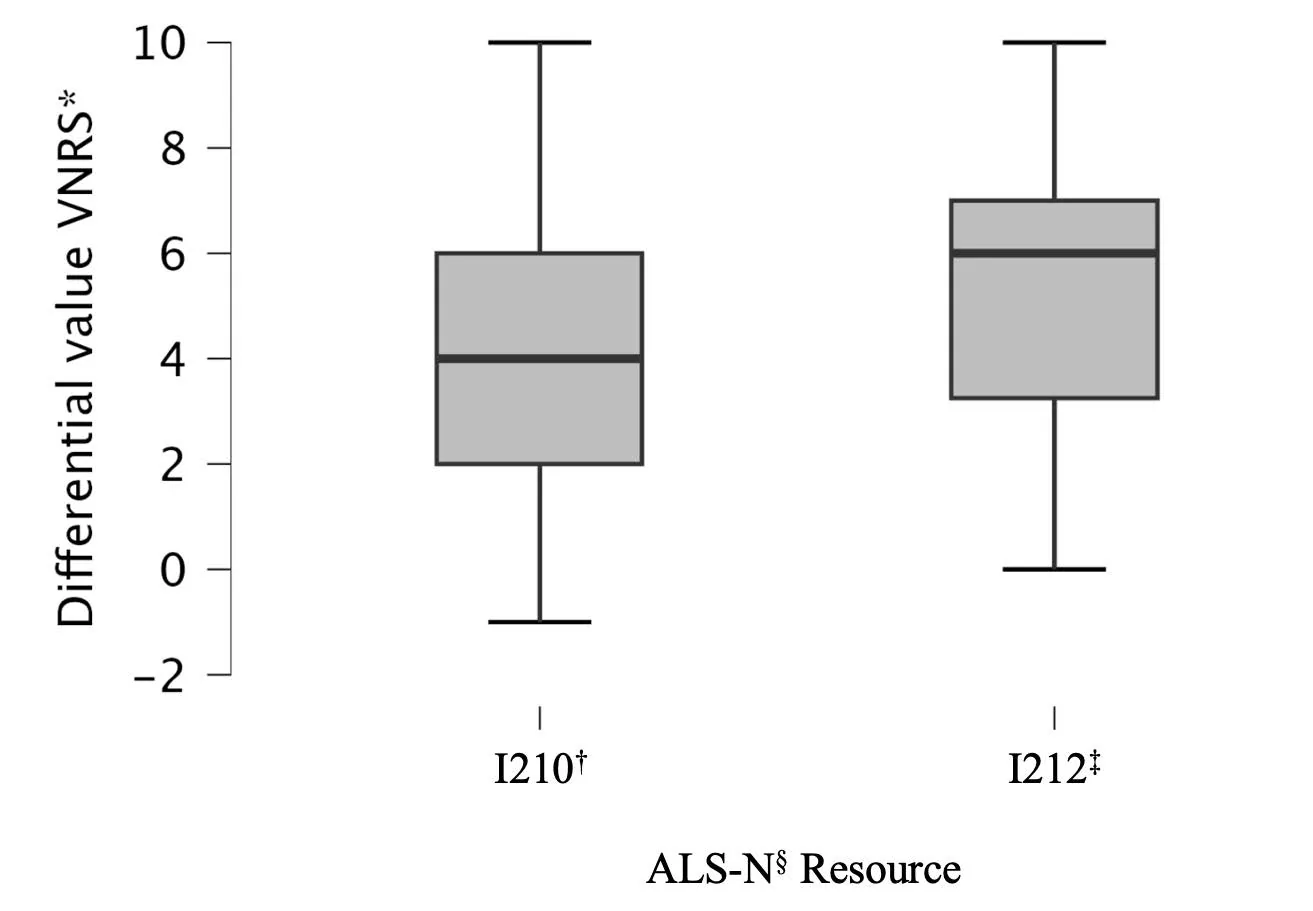
Box plot showing the differences between the Verbal Numerical Rating Scale scores, calculated as the final score minus the initial score, in the two advanced life support nursing units analysed

The bivariate analysis using the Wilcoxon signed-rank test between the initial and final VNRS values in the total sample resulted in W=12551, p < 0.001, and effect size r_bis = 0.998. Similarly, the Mann-Whitney U test between the reduction in VNRS in the two ALS-N units also showed a significant result (U= 2720.50, p < 0.005), with a negative effect size (r_bis negative at -0.258), indicating a greater reduction in pain for unit I212 (Figure 2).

**Table 2:** Frequencies of analgesia administered, grouped by families of active ingredients (n = 164). Palma de Mallorca, Balearic Islands, Spain, 2024

Combined analgesia	Frequency	Percentage	Valid percentage	Cumulative percentage
Analgesics	25	15.244	15.244	15.244
Opioids	92	56.098	56.098	71.341
Opioid and analgesic	35	21.341	21.341	92.683
Opioid and benzodiazepine	12	7.317	7.317	100.000
Total	164	100.000		

*VNRS = Verbal Numerical Rating Scale; ^†^I210 = Advanced life support nursing unit located in the capital, Palma de Mallorca; ^‡^I212 = Advanced life support nursing unit located in the town of Sóller; ^§^ALS-N = Advanced life support nursing unit

The correlation using Spearman’s rho between the duration of assistance (difference between the time of arrival at the incident and the time of transfer to the hospital) and the difference in VNRS (initial VNRS-final VNRS) was r_s_=0.141, p=0.036, under an alternative hypothesis of positive correlation. Univariate analysis between the four groups of drugs administered and the difference in VNRS, using the Kruskal-Wallis test, indicated significant differences between the grouped treatments (H(3)= 10.557, p=0.014) and between the two resources involved (H(1)=7.70, p=0.006), while it did not suggest differences between treatment groups and patient gender (H(1)=0.456, p=0.499). Dunn’s *post hoc* comparisons, with Bonferroni and Holm corrections, showed significant differences between non-opioid analgesics and opioids; the results are presented in Table 3. The Friedman test for repeated measurements between baseline and final VNS values showed χ2F (1)= 150.101, p<0.001. During the study period, no immediate adverse events were recorded after the administration of intravenous analgesia.

**Table 3:** Dunn’s *post hoc* comparisons between the different groups of analgesics administered (n = 164). Palma de Mallorca, Balearic Islands, Spain, 2024

Comparison	Z*	W_i_^†^	W_j_^‡^	r_rb_^§^	P^||^	p_Bonf_^¶^	p_Holm_**
Opioid analgesic	-2.850	55.340	85.592	0.370	0.004	0.026	0.026
Opioid analgesic + analgesic	-2.695	55.340	88.557	0.406	0.007	0.042	0.035
Opioid analgesic + benzodiazepine	-2.564	55.340	97.708	0.510	0.010	0.062	0.041
Opioid-opioid + analgesic	-0.317	85.592	88.557	0.034	0.751	1.000	1.000
Opioid-opioid + benzodiazepine	-0.839	85.592	97.708	0.154	0.402	1.000	1.000
Opioid + opioid analgesic + benzodiazepine	-0.581	88.557	97.708	0.100	0.561	1.000	1.000

*Z = Value in Dunn’s test; ^†^Wi = Mean rank of the first group being compared; ^‡^Wj= Mean rank of the second group being compared; ^§^rrb = Bisserial rank correlation based on individual Mann-Whitney contrasts; ^||^P = P value comparing z with the standard normal distribution; ^¶^pBonf = Adjusted p-value dividing the significance level by the number of multiple comparisons; **pHolm = p-value in multiple comparison, progressively adjusted to the significance level, starting with the lowest p-value

## Discussion

The literature shows that, in Europe, the presence of nurses leading certain models of care in pre-hospital emergencies is increasingly common; in countries such as Belgium, Finland, Sweden, the Netherlands, England, Wales, Italy, and Spain, the administration of certain drugs must be performed by accredited/registered nursing staff^11^ ^-^ ^13^. In Italy, some studies describe that nurses, with medical advice or through established protocols, administered analgesia in units of this type^14^. In Spain, the implementation of this model of ALS-N units is increasing, with operational variations depending on the region. 

In 2023, the MECC of SAMU 061 Baleares attended to 11,916 trauma cases that required the intervention of mobile care units. Of these, 8,667 patients were transported to hospital emergency services; according to the literature, approximately 70% may have experienced pain^1^ ^-^ ^5^. These data suggest that approximately 6,066 patients could have benefited from analgesic treatment, which can be administered by ALS-N units effectively and safely.

The assessment and treatment of post-traumatic pain have been studied for years^15^ and currently remain one of the cornerstones of the approach to traumatised patients^16^ ^-^ ^18^; inadequate pain management can delay return to work, reduce quality of life, and increase the risk of complications^19^. In a systematic review of the effectiveness of interventions performed by nursing staff in emergency departments, the administration of analgesia was identified as one of the most effective interventions for both reducing waiting time for administration and decreasing the pain perceived by patients. These findings are also supported by the results of another recent study^20^, in which nurses administered early analgesia to patients with probable wrist fractures. 

Currently, ultrasound-guided nerve blocks in patients with hip fractures by trained nursing staff are being studied and practiced^21^, an intervention also included in the most recent analgesia guidelines for these trauma patients^1^ ^,^ ^17^ ^,^ ^22^. It is worth highlighting the possibility of using the intranasal route for the administration of fentanyl as a fast and effective route, which has been shown in a study to be fast, safe, and effective in patients with hip fractures in hospital emergency departments^23^. 

In the literature search, there was a scarcity of studies evaluating the administration of analgesia by ALS-N units. An Italian study^14^ that evaluated the protocolisation of analgesia administration in the out-of-hospital setting described wide differences between the provinces analysed and a potential number of patients with pain who did not receive adequate treatment, in relation to the variability in the protocolisation of these procedures. The statistical analyses of our study showed a significant difference between the initial and final VNRS values in the sample (8.48±1.04 SD vs. 4.09±2.3 SD, W=12551.0, p<0.001).

These results can be compared to those of another Italian study^5^, in which, in ALS-N units that administered analgesia under medical prescription from the MECC, a significant improvement was observed between initial and final scores (8.36 ± 0.9 vs. 4.18 ± 2.2, t-test=6.23, p<0.001, n=25). In our study, the reduction was greater, = 4.39 ± 2.39 points. On the other hand, in the Italian study^5^, ALS-N reported using four drugs (paracetamol, ketorolac, fentanyl, and morphine), but did not perform a comparative analysis of the pain relief produced by each drug or their combinations. 

In our data, a greater reduction in VNRS was observed in unit I212 compared to I210. This finding could be related to the fact that I212 is located approximately 20 km from the capital (Palma de Mallorca), where most of the receiving hospitals are located, and to longer care and transport times (I210 = 42,8 ± 18,81 min vs. I212 = 64,38 ± 22,14 min), which means more time to achieve the desired analgesic effect. Therefore, the hypothesis was formulated that the increase in assistance and transport times could be positively correlated with a greater difference between the initial and final VNRS, allowing more time for the analgesia to take effect, especially in the case of non-opioid analgesics, whose onset of action may be slower. This hypothesis was supported by Spearman’s correlation, which showed a low but significant association (r_s_=0.141, p<0.05). 

Opioids are the most commonly used drugs for the treatment of severe acute musculoskeletal pain^24^ ^-^ ^25^. In our study, the administration of opioids alone or in combination with other drugs was associated with a greater reduction in perceived pain (H(3)= 10.557, p=0.014), a finding consistent with a recent systematic review of the efficacy of these treatments in the prehospital setting^26^. However, this study points out frequent limitations in the studies included, such as the absence of control groups, which reduces their methodological quality; this limitation is applicable to our study. 

On the other hand, there is a growing trend toward the use of multimodal strategies in the analgesic treatment of hospitalised trauma patients, with the aim of reducing the dose of opioids and, consequently, their adverse effects, without decreasing pain relief^27^. Considering that our study focused on patients with moderate or severe pain according to the VNRS (> 6 points), several international clinical guidelines recommend the combined intravenous administration of non-opioid and opioid analgesics as first-line treatment in these patients^17^ ^,^ ^22^, combinations that were frequently administered in our cohort.

Among the limitations of the study is the possibility of bias arising from the grouping of different classes of drugs administered, since the categories are broad and encompass different mechanisms of action. However, clinical guidelines indicate that, in the management of severe pain, the key seems to lie in a multimodal analgesic strategy, which includes the combination of drugs with different mechanisms of action^17^ ^,^ ^22^ ^,^ ^25^ ^,^ ^28^. To evaluate this effect, Dunn’s post hoc comparisons were performed between the treatment groups and the difference obtained in VNRS, with Holm and Bonferroni corrections, which showed the superiority of the groups that included opioids as the active ingredient over those that received only non-opioid analgesics. 

This finding can be contrasted with a systematic review and meta-analysis^2^, in which it was observed that the administration of nonsteroidal anti-inflammatory drugs produced a more effective reduction in pain 60 min after administration, compared to the administration of opioids in patients with musculoskeletal trauma treated in emergency departments. In our study, the duration of care was = 48,33’ ± 21,8 min, which does not allow us to evaluate this effect under the same conditions; however, the greater reduction in pain observed in the I212 unit might suggest a behaviour consistent with this trend.

In addition, there may have been a confounding bias because the number of doses and the concentration administered in each case were not verified, since some patients may have received more doses. However, this effect may have been mitigated thanks to the dosage adjusted to the patient’s approximate weight, prescribed by the regulating physician. It should also be noted that there may have been a selection bias among participants, since no randomisation procedure was applied due to the limited number of patients available. We chose to include all eligible patients during the study period in order to increase the representativeness of the sample and improve external validity, as well as to maximise the collection of relevant information.

Another limitation to be considered is the possible bias resulting from the subjectivity of the VNRS when measuring the pain perceived by patients. This scale may present significant interobserver variability^29^. In addition, this variability may be influenced by comprehension biases and cultural differences associated with the ethnicity and/or beliefs of patients according to their origin, factors described in a recent review^30^.

It should be noted that, despite these limitations, VNRS continues to be recommended by the scientific community, together with the visual analog scale^1^, as an indispensable tool for assessing pain in adult patients due to its speed and simplicity^31^ ^-^ ^32^. The use of multidimensional scales, such as the McGill Pain Questionnaire (MPQ) or the Brief Pain Inventory (BPI), could mitigate this limitation; however, they require more time to apply. The variability in the interval between both VNRS measurements, due to the lack of a record of the exact time of each assessment, may have produced a time measurement bias. In this study, the recorded time of arrival of the ALS-N unit at the incident site was considered the “initial assessment” and the time of transfer to the hospital was considered the “final assessment”, which approximates the time of both measurements. On the other hand, the absence of immediate adverse events in patients treated and medicated by the nursing staff of the two ALS-N units provides evidence of short-term safety. However, this finding only allows us to rule out early adverse events, such as anaphylactic reactions, which usually occur within the first 30 minutes^33^, or episodes of respiratory depression after analgesia and/or sedation, detectable early thanks to continuous monitoring, including capnography^34^, available on ALS-N monitors. Late adverse events, recorded in hospital records, were not considered, as their evaluation exceeded the specific objectives of this study.

## Conclusion

The administration of opioids to patients with trauma and moderate or severe pain by nursing staff in an out-of-hospital setting, with a prescription issued via telemedicine, produces a significant reduction in pain according to the Verbal Numerical Rating Scale (VNRS). The administration of medication by nursing staff in SAMU 061 advanced life support units, in addition to being effective, appears to be safe, with no adverse events reported in the short term. The increase in the interval from assistance start to arrival at the receiving hospital is associated with a greater reduction in perceived pain for these patients.

## Data Availability

All data generated or analysed during this study are included in this published article.
