# Supplementary material for: Analgesia in trauma patients treated by advanced life support nursing units: a retrospective pre-post intervention observational study
Source: Rev Lat Am Enfermagem. 2026 Aug 12;34:e4903. doi: 10.1590/1518-8345.7899.4903 (PMC13505608; doi:10.1590/1518-8345.7899.4903)
Supplement: Translation

# Translation: Analgesia em pacientes com traumatismo atendidos por unidades de suporte vital avançado de enfermagem: estudo observacional retrospectivo pré-pós-intervenção

**DOI:** 10.1590/1518-8345.7899.4904

**Authors:** Juan José García-Líndez, Ana María Uréndez-Ruíz, Juan Carlos Prieto-Gálvez, Jaume Ponce-Taylor, Francisco José Cereceda-Sánchez

**Affiliations:** 1 SAMU 061 Baleares, IB-Salut, Palma de Mallorca, Islas Baleares, Espanha.; 2 Universidad de las Islas Baleares, Facultad de Enfermería y Fisioterapia, Palma de Mallorca, Islas Baleares, Espanha.

**Keywords:** Enfermagem, Serviços Médicos de Emergência, Ambulâncias, Medição da Dor, Analgesia, Ferimentos e Lesões

## Abstract

**Objetivo:**

avaliar se a administração de diferentes grupos de analgésicos intravenosos a pacientes com traumatismo agudo e dor moderada ou intensa, em unidades de suporte vital avançado de enfermagem, reduziu significativamente a dor percebida.

**Método:**

estudo observacional retrospectivo e analítico, do tipo pré-pós-intervenção. Os dados foram coletados do sistema de informação e dos prontuários clínicos. Foram analisados, por meio de estatística descritiva e testes não paramétricos, o nível de dor no início e ao final da assistência, sua associação com os fármacos administrados e os tempos assistenciais.

**Resultados:**

foram avaliados os dados de 164 pacientes. A idade média foi de 54±21,49 anos e 48,78% eram mulheres. A pontuação inicial na Escala Numérica Verbal foi de = 8,48±1,04 pontos, enquanto ao final da assistência foi de = 4,09±2,32 pontos; a redução foi de = 4,39±2,39 pontos, com diferença estatisticamente significativa entre ambos os momentos (p < 0,001). Os tratamentos com opioides mostraram maior eficácia analgésica do que os analgésicos não opioides (p=0,014).

**Conclusão:**

a administração de analgesia pela enfermagem em unidades de suporte vital avançado no âmbito extra-hospitalar alcança uma redução da dor percebida em traumatismos agudos. Os opioides, isolados ou combinados com analgésicos não opioides, associam-se a uma maior redução da dor.

## Destaques

**(1)** A analgesia intravenosa reduz significativamente a dor em pacientes com traumatismo. **(2)** Os opioides, isolados ou combinados com analgésicos não opioides, produzem maior alívio da dor. **(3)** A unidade de suporte vital avançado de enfermagem é segura e eficaz na administração de analgesia. **(4)** A administração de analgesia intravenosa não se associou a eventos adversos em curto prazo. **(5)** O prolongamento do tempo assistencial se correlaciona com maior redução da dor traumática.

## Introdução

Na Europa, estima-se que cerca de 38 milhões de pessoas são atendidas anualmente nos serviços de urgência por um evento traumático^1^. A dor aguda constitui um motivo de consulta frequente entre os pacientes que sofreram um traumatismo. A prevalência documentada gira em torno de 70% no âmbito pré-hospitalar, e a literatura sugere que pode chegar a 90%^2^ ^-^ ^5^. Em determinados países europeus, documentou-se que apenas entre 14% e 32% dos pacientes com dor moderada a intensa receberam analgesia durante a assistência pré-hospitalar^1^. Em um estudo realizado nos Estados Unidos da América (EUA), indicou-se que até 50% dos pacientes com fraturas ósseas não haviam recebido analgesia nos serviços de urgência^6^. Na fase de atenção pré-hospitalar dos traumatismos graves, recomenda-se o alívio precoce e adequado da dor aguda, com o objetivo de facilitar o transporte do paciente ao hospital, garantir seu conforto e reduzir os efeitos prejudiciais da dor e do estresse associado^7^. Diversos estudos indicam que o uso de um protocolo de analgesia iniciado pela enfermagem, tanto no âmbito extra-hospitalar quanto nos serviços de urgência, diminui o tempo até o manejo da dor, reduz sua intensidade e aumenta a satisfação do paciente^6^ ^,^ ^8^ ^-^ ^9^.

Na avaliação da dor, as escalas unidimensionais, como a Escala Numérica Verbal (ENV) ou a escala visual analógica, estão entre as mais utilizadas^10^, devido à facilidade de uso e à sua padronização^7^. A ENV é a escala empregada atualmente nos pacientes atendidos pelo SAMU 061 Baleares.

Há mais de uma década, as unidades de suporte vital avançado de enfermagem (SVAE) são reconhecidas legalmente como recursos assistenciais avançados de emergência no âmbito extra-hospitalar na Espanha. Trata-se de recursos liderados pela enfermagem, dotados do material necessário para fornecer a atenção requerida de forma comparável a uma unidade de suporte vital avançado (SVA) com médico. Os SVAE atuam com respaldo e assessoramento, e recebem prescrição farmacológica quando necessário pelos médicos reguladores da Central de Coordenação de Urgências Médicas (CCUM) do SAMU 061 Baleares, por meio de telemedicina. Esse modelo de recursos assistenciais começou a ser implementado no sistema sanitário espanhol há três décadas, inicialmente sem um marco normativo específico. Desde então, evoluiu de forma heterogênea entre as diferentes comunidades autônomas. Nesta comunidade autônoma, desde 2018 há unidades desse tipo; atualmente existem duas: uma na cidade de Palma de Mallorca (I210) e outra na população de Sóller (I212).

Esses recursos SVAE são compostos por um técnico em emergências sanitárias e uma enfermeira. Esta última deve contar com mais de um ano de experiência prévia no serviço, formação credenciada em SVA, formação específica em SVAE ministrada pela área de formação do SAMU 061 Baleares e ter realizado plantões supervisionados em unidade SVAE antes de sua incorporação, de acordo com as diretrizes do grupo de trabalho de SVAE da *Sociedad Española de Medicina de Urgencias y Emergencias* (SEMES).

O objetivo principal do estudo foi avaliar se a administração de diferentes grupos de analgésicos intravenosos a pacientes com traumatismo agudo e dor moderada ou intensa, em unidades de suporte vital avançado de enfermagem, reduziu significativamente a dor percebida. Os objetivos secundários foram: (i) descrever a frequência de eventos adversos após a administração de analgesia; (ii) determinar se os diferentes grupos de analgésicos utilizados apresentavam eficácia similar para reduzir a dor; (iii) avaliar se existiam diferenças na redução da dor percebida conforme os tempos de assistência e de transporte entre as duas unidades de suporte vital de enfermagem estudadas.

## Métodos

### Tipo de estudo e local

Estudo observacional retrospectivo, analítico e unicêntrico, do tipo pré-pós-intervenção. Foi realizado no âmbito das urgências extra-hospitalares na cidade de Palma de Mallorca e na população de Sóller, na Ilha de Mallorca, Ilhas Baleares, Espanha.

### População e período de estudo

Pacientes adultos assistidos após um traumatismo entre janeiro de 2019 e dezembro de 2023 por alguma das duas SVAE participantes. Nos casos em que se requeria medicação, a enfermeira realizava punção venosa periférica e solicitava, por meio de telemedicina, o esquema de analgesia intravenosa ao médico regulador.

### Participantes do estudo e critérios de seleção

A amostra foi obtida por meio de uma amostragem não probabilística por conveniência. Foram incluídos inicialmente todos os pacientes adultos atendidos por uma SVAE após um traumatismo registrado no sistema de informação durante o período de estudo. Os critérios de inclusão foram: pacientes com mais de 14 anos atendidos com algum dos códigos CIE relacionados a traumatismos e com critérios clínicos sugestivos de fratura; dispor de registro da escala numérica verbal da dor no início da assistência (ENV inicial), com pontuação ≥ 6, e de registro na chegada ao centro hospitalar (ENV final). Os critérios de exclusão foram: casos sem registro completo da escala numérica da dor (ENV inicial e final); casos em que não foi administrada analgesia intravenosa; pacientes pediátricos (menores de 14 anos); e pacientes sem registro do tempo de assistência (diferença entre o horário de chegada ao incidente e o horário de transferência ao hospital de destino). 

### Variáveis do estudo

As variáveis independentes foram: sociodemográficas (sexo e idade), analgesia administrada, valor inicial da escala numérica de dor, código da unidade assistencial e tempo de assistência. As variáveis dependentes foram: valor da escala numérica verbal da dor ao final da assistência e número de eventos adversos registrados após a administração de analgesia.

### Coleta e análise dos dados

Os dados foram coletados do sistema de informação SENECA^©^ da CCUM e das fichas assistenciais do SAMU 061 Baleares, e foram tratados de forma anonimizada. A análise foi realizada com o programa *Just Another Statistical Program* (JASP). As variáveis quantitativas foram descritas por meio de média (), desvio-padrão (DP) e mediana; as variáveis categóricas, por meio de frequências e porcentagens. Antes de realizar as inferências estatísticas, avaliou-se a normalidade da distribuição das variáveis quantitativas por meio do teste de Shapiro-Wilk. Como se constatou a ausência de normalidade, empregaram-se testes não paramétricos: teste de Wilcoxon (W), U de Mann-Whitney (U), teste de Friedman (χ^2^F), coeficiente *rho* de Spearman (r_s_) e teste de Kruskal-Wallis (H). Além disso, quando se identificaram diferenças significativas, aplicou-se o teste *post hoc* não paramétrico de Dunn, com correções de Holm e Bonferroni. As associações entre variáveis foram avaliadas por meio de testes de hipótese com nível de significância p<0,05. A fim de reduzir o viés de relato, apresentam-se também os resultados não significativos (p>0,05). Para efetuar as inferências estatísticas e dada a diversidade de tratamentos administrados, estes foram agrupados em quatro categorias segundo a família de princípios ativos e as combinações realizadas durante a assistência: analgésicos não opioides (paracetamol, metamizol, dexketoprofeno), opioides (cloreto mórfico, fentanil, meperidina), opioides combinados com benzodiazepínicos (diazepam, midazolam). 

### Aspectos éticos

O presente estudo se ajustou aos princípios da Declaração de Helsinque e contou com a autorização do Comitê de Ética da Pesquisa em Saúde das Ilhas Baleares (IB5553/24 PI).

## Resultados

O número total de pacientes incluídos na análise durante o período de estudo é apresentado na Figura 1.

**Figura 1:** Fluxograma do recrutamento da amostra analisada

*SVAE = Suporte Vital Avançado de Enfermagem; ^†^ENV = Escala Numérica Verbal

Na Tabela 1, apresentam-se as estatísticas descritivas da amostra e as principais variáveis do estudo.

A idade média dos pacientes foi de 54 ± 21,49 anos, e 48,78% eram mulheres (n=80). Do total, 74,39% (n=122) dos atendimentos foram realizados pela unidade I210. A pontuação na ENV inicial foi de x ® = 8,48 ± 1,04 pontos, enquanto na ENV final foi de x ® = 4,09 ± 2,32 pontos. A redução após a administração do tratamento foi de x ® = 4,39 ± 2,39 pontos. Em 5,48% dos pacientes, não se observou redução da dor percebida (n=9), apesar da administração de analgesia.

**Tabela 1:** Estatísticas descritivas das variáveis analisadas segundo o recurso de suporte vital avançado de enfermagem (SVAE*), com testes de aderência (n = 164). Palma de Mallorca, Ilhas Baleares, Espanha, 2024

Variáveis por recurso SVAE*	Idade		Tempo assistência		ENV^†^ inicial		ENV^†^ final		Diferença ENV^†^	
	I210^‡^	I212^§^	I210^‡^	I212^§^	I210^‡^	I212^§^	I210^‡^	I212^§^	I210^‡^	I212^§^
Moda	52,85	65,48	42,58	64,27	9,97	8,0	5,42	2,19	3,79	6,05
Mediana	52,0	61,5	41,63	64,34	8,0	8,0	4,0	3,0	4,0	6,0
Desvio-padrão	20,96	22,26	18,81	22,14	1,41	1,41	2,29	2,20	2,35	2,32
Coeficiente de variação	0,40	0,37	0,43	0,34	0,16	0,16	0,52	0,67	0,53	0,44
Intervalo interquartílico	33,75	35,25	22,44	23,14	2,0	2,0	3,75	3,0	4,0	3,75
Assimetria	0,08	-0,15	0,81	0,19	-0,33	-0,38	0,03	0,62	0,29	-0,37
Erro-padrão da assimetria	0,21	0,36	0,21	0,36	0,21	0,36	0,21	0,36	0,21	0,36
Curtose	-0,94	-1,01	1,4	-0,09	-1,17	-0,98	-0,55	-0,28	-0,18	-0,38
Erro-padrão da curtose	0,43	0,71	0,43	0,71	0,43	0,71	0,43	0,71	0,43	0,71
Shapiro-Wilk	0,97	0,96	0,96	0,98	0,84	0,82	0,96	0,92	0,96	0,95
Valor de p Shapiro-Wilk	0,01	0,22	0,00	0,67	<,001	<,001	0,004	0,008	0,006	0,10
Amplitude	80,0	80,0	104,6	91,4	4,0	4,0	10,0	8,0	11,0	10,0
Mínimo	15,0	19,0	8,18	21,38	6,0	6,0	0,00	0,00	-1,00	0,00
Máximo	95,0	99,0	112,8	112,8	10,0	10,0	10,0	8,0	10,0	10,0
Primeiro quartil	35,25	43,25	30,16	52,32	8,0	8,0	2,25	2,0	2,0	3,25
Terceiro quartil	69,0	78,5	52,6	75,4	10,0	10,0	6,0	5,0	6,0	7,0

*SVAE = Suporte Vital Avançado de Enfermagem; ^†^ENV = Escala Numérica Verbal; ^‡^I210 = Recurso de suporte vital avançado de enfermagem localizado na capital, Palma de Mallorca; ^§^I212 = Recurso de suporte vital avançado de enfermagem localizado na população de Sóller

Na Tabela 2, apresentam-se as frequências dos tratamentos administrados, agrupados por família de princípios ativos.

A análise bivariada por meio do teste de postos sinalizados de Wilcoxon entre os valores iniciais e finais da ENV na amostra total resultou em W=12551, p < 0,001 e tamanho de efeito r_bis 0,998. Da mesma forma, o teste U de Mann-Whitney entre a redução da ENV nas duas unidades SVAE também mostrou resultado significativo (U= 2720,50, p < 0,005), com tamanho de efeito negativo (r_bis negativo de -0,258), o que indica maior redução da dor na unidade I212 (Figura 2).

**Tabela 2:** Frequências da analgesia administrada, agrupada por famílias de princípios ativos (n = 164). Palma de Mallorca, Ilhas Baleares, Espanha, 2024

Analgesia agrupada	Frequência	Porcentagem	Porcentagem válida	Porcentagem acumulada
Analgésicos	25	15.244	15.244	15.244
Opioides	92	56.098	56.098	71.341
Opioide e analgésico	35	21.341	21.341	92.683
Opioide e benzodiazepínico	12	7.317	7.317	100.000
Total	164	100.000		

**Figura 2:** Diagrama de caixa com as diferenças entre os valores da Escala Numérica Verbal, calculadas como registro inicial menos o final, nos dois recursos de suporte vital avançado de enfermagem analisados

*ENV = Escala Numérica Verbal; ^†^I210 = Recurso de suporte vital avançado de enfermagem localizado na capital, Palma de Mallorca; ^‡^I212 = Recurso de suporte vital avançado de enfermagem localizado na população de Sóller; ^§^SVAE = Suporte vital avançado de enfermagem

A correlação por meio do rho de Spearman entre o tempo de assistência (diferença entre o horário de chegada ao incidente e o horário de transferência ao hospital) e a diferença na ENV (ENV inicial-ENV final) foi r_s_=0,141, p=0,036, sob uma hipótese alternativa de correlação positiva. A análise univariada entre os quatro grupos de fármacos administrados e a diferença na ENV, por meio do teste de Kruskal-Wallis, indicou diferenças significativas entre os tratamentos agrupados (H(3)= 10,557, p=0,014) e entre os dois recursos envolvidos (H(1)=7,70, p=0,006), enquanto não sugeriu diferenças entre os grupos de tratamento e o sexo dos pacientes (H(1)=0,456, p=0,499). As comparações *post hoc* de Dunn, com correções de Bonferroni e Holm, mostraram diferenças significativas entre os analgésicos não opioides e os opioides; os resultados são apresentados na Tabela 3. O teste de Friedman para medições repetidas entre os valores inicial e final da ENV mostrou χ^2^F (1)= 150,101, p<0,001. Durante o período de estudo, não foram registrados eventos adversos imediatos após a administração de analgesia intravenosa.

**Tabela 3:** Comparações *post hoc* de Dunn entre os diferentes grupos de analgésicos administrados (n = 164). Palma de Mallorca, Ilhas Baleares, Espanha, 2024

Comparação	Z*	W_i_^†^	W_j_^‡^	r_rb_^§^	P^||^	p_Bonf_^¶^	p_Holm_**
Analgésico-opioide	-2.850	55.340	85.592	0.370	0.004	0.026	0.026
Analgésico-opioide+ analgésico	-2.695	55.340	88.557	0.406	0.007	0.042	0.035
Analgésico-opioide+benzodiazepínico	-2.564	55.340	97.708	0.510	0.010	0.062	0.041
Opioide-opioide+ analgésico	-0.317	85.592	88.557	0.034	0.751	1.000	1.000
Opioide-opioide+ benzodiazepínico	-0.839	85.592	97.708	0.154	0.402	1.000	1.000
Opioide+analgésico-opioide+benzodiazepínico	-0.581	88.557	97.708	0.100	0.561	1.000	1.000

*Z = Valor z no teste de Dunn; ^†^W_i_ = Posto médio do primeiro grupo que se compara; ^‡^W_j_ = Posto médio do segundo grupo comparado; ^§^r_rb_ = Correlação bisserial de postos baseada em contrastes individuais de Mann-Whitney; ^||^P = Valor de p comparando z com a distribuição normal padrão; ^¶^p_Bonf_ = Valor de p ajustado dividindo o nível de significância pelo número de comparações múltiplas; **p_Holm_ = Valor de p na comparação múltipla, ajustado progressivamente ao nível de significância, começando pelo menor valor de p

## Discussão

A literatura mostra que, no continente europeu, a presença da enfermagem liderando determinados modelos assistenciais nas emergências pré-hospitalares está cada vez mais difundida; em países como Bélgica, Finlândia, Suécia, Países Baixos, Inglaterra, País de Gales, Itália ou Espanha, para administrar determinados fármacos, exige-se que a administração seja realizada por equipe de enfermagem credenciada/registrada^11^ ^-^ ^13^. Na Itália, alguns estudos descrevem que a enfermagem, com assessoramento médico ou por meio de protocolos estabelecidos, administrava analgesia em unidades desse tipo^14^. Na Espanha, está aumentando a implementação desse modelo de recursos SVAE, com variações operacionais em função da comunidade autônoma. 

Na CCUM do SAMU 061 Baleares, em 2023, foram atendidos 11.916 casos de traumatismos que requereram a intervenção de unidades móveis assistenciais. Desses, 8.667 pacientes foram transportados para serviços de urgência hospitalar; segundo a literatura, aproximadamente 70% poderiam ter apresentado dor^1^ ^-^ ^5^. Esses dados sugerem que aproximadamente 6.066 pacientes poderiam ter se beneficiado de tratamento analgésico, tratamento que pode ser administrado pelas unidades SVAE de forma eficaz e segura.

A avaliação e o tratamento da dor pós-traumática vêm sendo objeto de estudo há anos^15^ e, atualmente, continuam sendo uma das pedras angulares da abordagem do paciente traumatizado^16^ ^-^ ^18^; o manejo inadequado da dor pode atrasar o retorno ao trabalho, reduzir a qualidade de vida e aumentar o risco de complicações^19^. Em uma revisão sistemática sobre a efetividade das intervenções realizadas por equipe de enfermagem nos serviços de urgência^9^, a administração de analgesia foi identificada como uma das intervenções mais eficazes tanto para reduzir o tempo de espera até a administração quanto para diminuir a dor percebida pelos pacientes. Esses achados também são apoiados pelos resultados de outro estudo recente^20^, no qual as enfermeiras administravam analgesia precoce a pacientes com prováveis fraturas de punho. 

Atualmente, inclusive, está sendo estudada e praticada a realização de bloqueios nervosos guiados por ultrassom em pacientes com fraturas de quadril por equipe de enfermagem treinado^21^, intervenção também contemplada nas diretrizes mais recentes de analgesia para esses pacientes traumatizados^1^ ^,^ ^17^ ^,^ ^22^. Deve-se destacar a possibilidade de utilizar a via intranasal para a administração de fentanil como uma via rápida e eficaz, a qual demonstrou, em um estudo, que seu uso em pacientes com fratura de quadril, no âmbito das urgências hospitalares, foi rápido, seguro e eficaz^23^. 

Na busca bibliográfica realizada, observou-se escassez de estudos que avaliassem a administração de analgesia por unidades SVAE. Em um estudo italiano^14^ que avaliou a protocolização da administração de analgesia no meio extra-hospitalar, descreveram-se amplas diferenças entre as províncias analisadas e um número potencial de pacientes com dor que não recebiam tratamento adequado, em relação à variabilidade na protocolização desses procedimentos. As análises estatísticas do nosso estudo evidenciaram uma diferença significativa entre os valores iniciais e finais da ENV no conjunto da amostra (8,48±1,04 DP vs. 4,09±2,3 DP, W=12551,0, p<0,001).

Esses resultados podem ser comparados aos de outro estudo italiano^5^, no qual, em unidades SVAE que administravam analgesia sob prescrição médica a partir da CCUM, observou-se uma melhora significativa entre os valores iniciais e finais (8,36 ± 0,9 vs. 4,18 ± 2,2, t-test=6,23, p<0,001, n=25). Em nosso estudo, a redução foi superior, = 4,39 ± 2,39 pontos. Por outro lado, no trabalho italiano^5^, as SVAE relataram ter utilizado quatro fármacos (paracetamol, cetorolaco, fentanil e morfina), mas não realizaram análise comparativa do alívio da dor produzido por cada fármaco nem por suas combinações. 

Em nossos dados, observou-se maior redução da ENV na unidade I212 em comparação com a I210. Esse achado poderia relacionar-se ao fato de a I212 estar localizada a aproximadamente 20 km da capital (Palma de Mallorca), onde se encontram a maioria dos hospitais receptores, e a um maior tempo de assistência e transporte (I210 = 42,8 ± 18,81 min vs. I212 = 64,38 ± 22,14 min), o que poderia ter permitido mais tempo para alcançar o efeito analgésico desejado. Por isso, formulou-se a hipótese de que o aumento do tempo de assistência e transporte poderia correlacionar-se positivamente com um maior diferencial entre a ENV inicial e final, ao permitir mais tempo para que a analgesia alcance seu efeito, especialmente no caso de analgésicos não opioides, cujo início de ação pode ser mais lento. Essa hipótese foi sustentada pela correlação de Spearman, que mostrou uma associação baixa, porém significativa (r_s_=0,141, p<0,05). 

Os opioides são os fármacos mais utilizados para o tratamento da dor aguda musculoesquelética intensa^24^ ^-^ ^25^. Em nosso estudo, a administração de opioides de forma isolada ou em combinação com outros fármacos associou-se a uma maior redução da dor percebida (H(3)= 10.557, p=0,014), achado consistente com uma revisão sistemática recente sobre a eficácia desses tratamentos no âmbito pré-hospitalar^26^. No entanto, esse trabalho aponta limitações frequentes nos estudos incluídos, como a ausência de grupos controle, o que reduz sua qualidade metodológica; essa limitação é aplicável ao nosso estudo. 

Por outro lado, observa-se uma tendência crescente ao uso de estratégias multimodais no tratamento analgésico de pacientes traumatizados hospitalizados, com o objetivo de reduzir a dose de opioides e, consequentemente, seus efeitos adversos, sem diminuir o alívio da dor^27^. Considerando que nosso estudo se concentrou em pacientes com dor moderada ou intensa segundo a ENV (> 6 pontos), várias diretrizes clínicas internacionais recomendam a administração intravenosa combinada de analgésicos não opioides e opioides como tratamento de primeira linha nesses pacientes^17^ ^,^ ^22^, combinações que foram administradas com frequência em nossa coorte.

Entre as limitações do estudo, encontra-se a possibilidade de ter ocorrido um viés decorrente do agrupamento dos diferentes grupos de fármacos administrados, uma vez que as categorias são amplas e reúnem mecanismos de ação distintos. No entanto, as diretrizes clínicas assinalam que, no manejo da dor intensa, a chave parece residir em uma estratégia analgésica multimodal, que inclui a combinação de fármacos com diferentes mecanismos de ação^17^ ^,^ ^22^ ^,^ ^25^ ^,^ ^28^. Para avaliar esse efeito, foram realizadas comparações *post hoc* de Dunn entre os grupos de tratamento e a diferença obtida na ENV, com correções de Holm e Bonferroni, que evidenciaram superioridade dos grupos que incluíam opioides como princípio ativo em relação aos que receberam apenas analgésicos não opioides. 

Esse achado pode ser contrastado com uma revisão sistemática e metanálise^2^, na qual se observou que a administração de anti-inflamatórios não esteroides produzia uma redução da dor mais eficaz aos 60 min após a administração, em comparação com a administração de opioides em pacientes com traumatismos musculoesqueléticos atendidos em serviços de urgência. Em nosso estudo, o tempo de assistência foi de = 48,33’ ± 21,8 min, de modo que não permite avaliar esse efeito nas mesmas condições; no entanto, a maior redução da dor observada na I212 poderia sugerir um comportamento compatível com essa tendência.

Além disso, pode ter havido um viés de confusão por não se ter verificado o número de doses e a concentração administradas em cada caso, uma vez que alguns pacientes poderiam ter recebido mais doses. No entanto, esse efeito pode ter sido mitigado graças à dosagem ajustada ao peso aproximado do paciente, prescrita pelo médico regulador. Deve-se destacar, ainda, o possível viés de seleção dos participantes, uma vez que não foi aplicado nenhum procedimento de randomização devido ao número limitado de pacientes disponíveis. Por isso, optou-se por incluir todos os pacientes elegíveis durante o período do estudo, com o objetivo de aumentar a representatividade da amostra e melhorar a validade externa, bem como maximizar a obtenção de informação relevante.

Outra limitação a ser considerada é o possível viés decorrente da subjetividade da ENV ao medir a dor percebida pelos pacientes. Essa escala pode apresentar variabilidade interobservador significativa^29^. Além disso, essa variabilidade pode ser influenciada por vieses de compreensão e por diferenças culturais associadas à etnia e/ou às crenças dos pacientes conforme sua origem, fatores descritos em uma revisão recente^30^.

Cabe ressaltar que, apesar dessas limitações, a ENV continua sendo recomendada pela comunidade científica, juntamente com a escala visual analógica^1^, como ferramenta imprescindível para a avaliação da dor em pacientes adultos por sua rapidez e simplicidade^31^ ^-^ ^32^. O uso de escalas multidimensionais, como a *McGill Pain Questionnaire* (MPQ) ou o *Brief Pain Inventory* (BPI), poderia atenuar essa limitação^19^; no entanto, requerem mais tempo para sua aplicação. A variabilidade do intervalo entre ambas as medições da ENV, por não se dispor do registro do momento exato de cada avaliação, pode ter produzido um viés de mensuração do tempo. Neste estudo, considerou-se como “avaliação inicial” o horário registrado de chegada da SVAE ao local e como “avaliação final” o horário de transferência ao hospital, o que aproxima o momento de ambas as medições. Por outro lado, a ausência de eventos adversos imediatos nos pacientes atendidos e medicados pela equipe de enfermagem das duas unidades de SVAE fornece evidência de segurança em curto prazo. No entanto, esse achado apenas permite descartar eventos adversos de início precoce, como reações anafiláticas, que costumam se apresentar nos primeiros 30 min^33^, ou episódios de depressão respiratória após analgesia e/ou sedação, detectáveis precocemente graças à monitorização contínua, incluindo a capnografia^34^, disponível nos monitores das SVAE. Os eventos adversos tardios, registrados no prontuário hospitalar, não foram considerados, pois sua avaliação excedia os objetivos específicos do presente trabalho.

## Conclusão

A administração de opioides a pacientes com traumatismo e dor moderada ou intensa por equipe de enfermagem no âmbito extra-hospitalar, com prescrição médica por meio de telemedicina, produz uma redução significativa da dor segundo a Escala Numérica Verbal (ENV). A administração de medicação pela equipe de enfermagem em unidades de suporte vital avançado de enfermagem do SAMU 061, além de eficaz, parece segura, ao não se registrarem eventos adversos em curto prazo. O prolongamento do intervalo desde o início da assistência até a chegada ao hospital receptor associa-se a uma maior redução da dor percebida nesses pacientes.

## Data Availability

Todos os dados gerados ou analisados durante este estudo estão incluídos neste artigo publicado.

# Translation: Analgesia en pacientes con traumatismo asistidos por unidades de soporte vital avanzado de enfermería: estudio observacional retrospectivo pre-postintervención

**DOI:** 10.1590/1518-8345.7899.4902

**Authors:** Juan José García-Líndez, Ana María Uréndez-Ruíz, Juan Carlos Prieto-Gálvez, Jaume Ponce-Taylor, Francisco José Cereceda-Sánchez

**Affiliations:** 1 SAMU 061 Baleares, IB-Salut, Palma de Mallorca, Islas Baleares, España.; 2 Universidad de las Islas Baleares, Facultad de Enfermería y Fisioterapia, Palma de Mallorca, Islas Baleares, España.

**Keywords:** Enfermería, Servicios Médicos de Urgencia, Ambulancias, Dimensión del Dolor, Analgesia, Heridas y Lesiones

## Abstract

**Método:**

estudio observacional retrospectivo y analítico, de tipo pre-postintervención. Los datos se recopilaron del sistema de información y de las historias clínicas. Se analizaron mediante estadística descriptiva y pruebas no paramétricas el nivel del dolor al inicio y al final de la asistencia, su asociación con los fármacos administrados y los tiempos asistenciales.

**Resultados:**

se evaluaron los datos de 164 pacientes. La edad media fue de 54±21,49 años y el 48,78% era mujeres. La puntuación inicial en la escala numérica verbal fue de = 8,48±1,04 puntos, mientras que al final de la asistencia fue de = 4,09±2,32 puntos; la reducción media fue de = 4,39±2,39 puntos con una diferencia estadísticamente significativa entre ambos momentos (p < 0,001). Los tratamientos con opioides mostraron mayor eficacia analgésica que los analgésicos no opioides (p=0,014).

**Conclusión:**

La administración de analgesia por enfermería en unidades de soporte vital avanzado en el ámbito extrahospitalario logra una reducción del dolor percibido en traumatismos agudos. Los opioides, solos o combinados con analgésicos no opioides, se asocian con una mayor reducción del dolor.

## Destacados

**(1)** La analgesia intravenosa reduce significativamente el dolor en pacientes con traumatismo. **(2)** Los opioides, solos o combinados con analgésicos no opioides, producen mayor alivio del dolor. **(3)** La unidad de soporte vital avanzado de enfermería es segura y efectiva en la administración de analgesia. **(4)** La administración de analgesia intravenosa no se asoció con eventos adversos a corto plazo. **(5)** La prolongación del tiempo asistencial se correlaciona con una mayor reducción del dolor traumático.

## Introducción

En Europa se estima que cerca de 38 millones de personas son atendidas anualmente en los servicios de urgencias por un evento traumático^1^. El dolor agudo constituye un motivo de consulta frecuente entre los pacientes que han sufrido un traumatismo. La prevalencia documentada ronda el 70% en el ámbito prehospitalario y se ha sugerido que puede alcanzar el 90%^2^ ^-^ ^5^. En determinados países europeos se ha documentado que solo entre el 14% y el 32% de los pacientes con dolor moderado a intenso recibieron analgesia durante la asistencia prehospitalaria^1^. En un estudio efectuado en Estados Unidos de América (EE. UU.) se indicó que hasta el 50% de los pacientes con fracturas óseas no habían recibido analgesia en los servicios de urgencias^6^. En la fase de atención prehospitalaria de los traumatismos graves se recomienda el alivio temprano y adecuado del dolor agudo, con el fin de facilitar el traslado del paciente al hospital, garantizar su comodidad y reducir de los efectos perjudiciales del dolor y del estrés asociado^7^. Diversos estudios indican que el uso de un protocolo de analgesia iniciado por enfermería, tanto en el ámbito extrahospitalario y en los servicios de urgencias, disminuye el tiempo hasta el manejo del dolor, reduce su intensidad y aumenta la satisfacción del paciente^6^ ^,^ ^8^ ^-^ ^9^. 

En la evaluación del dolor, las escalas unidimensionales, como la escala numérica verbal (ENV) o la escala visual analógica, se encuentran entre las más utilizadas^10^, debido a la facilidad de uso y su estandarización^7^. La ENV es la escala empleada actualmente en los pacientes atendidos por el SAMU 061 Baleares.

Desde hace más de una década, las unidades de soporte vital avanzado de enfermería (SVAE) están reconocidas legalmente como recursos asistenciales avanzados de emergencia en el ámbito extrahospitalario en España. Se trata de recursos liderados por enfermería, dotados del material necesario para proporcionar la atención requerida de forma comparable a una unidad de soporte vital avanzado (SVA) con médico. Los SVAE actúan con respaldo y asesoramiento, y reciben prescripción farmacológica cuando es necesario por los médicos reguladores de la Central de Coordinación de Urgencias Médicas (CCUM) del SAMU 061 Baleares, mediante telemedicina. Este modelo de recursos asistenciales comenzó a implementarse en el sistema sanitario español hace tres décadas, inicialmente sin un marco normativo específico. Desde entonces, ha evolucionado de forma heterogénea entre las diferentes comunidades autónomas. En esta comunidad autónoma, desde 2018 se dispone de unidades de este tipo; en la actualidad existen dos: una en la ciudad de Palma de Mallorca (I210) y otra en la Población de Sóller (I212). 

Estos recursos SVAE están integrados por un técnico en emergencias sanitarias y una enfermera. Esta última debe contar con más de un año de experiencia previa en el servicio, formación acreditada en SVA, formación específica SVAE impartida por el área de formación del SAMU 061 Baleares y haber realizado guardias tutorizadas en unidad SVAE antes de su incorporación, de acuerdo con las directrices del grupo de trabajo de SVAE de la Sociedad Española de Medicina de Urgencias y Emergencias (SEMES). 

El objetivo principal del estudio fue evaluar si la administración de diferentes grupos de analgésicos intravenosos, en unidades de soporte vital avanzado de enfermería, a pacientes con traumatismo agudo y dolor moderado o intenso, reducía significativamente el dolor percibido. Los objetivos secundarios fueron: (i) describir la frecuencia de eventos adversos tras la administración de analgesia; (ii) determinar si los diferentes grupos de analgésicos utilizados presentaban una eficacia similar para reducir el dolor; (iii) evaluar si existían diferencias en la reducción del dolor percibido según los tiempos de asistencia y de traslado entre las dos unidades de soporte vital de enfermería estudiadas.

## Métodos

### Tipo de estudio y lugar

Estudio observacional retrospectivo, analítico y unicéntrico, de tipo pre-postintervención, realizado en el ámbito de las urgencias extrahospitalarias en la ciudad de Palma de Mallorca y en la Población de Sóller, en la Isla de Mallorca, Baleares, España.

### Población y periodo de estudio

Pacientes adultos asistidos tras un traumatismo entre enero de 2019 y diciembre de 2023 por alguna de las dos SVAE participantes. En los casos en que se requerían medicación, la enfermera canalizaba una vía venosa periférica y solicitaba al médico regulador, mediante telemedicina, la pauta de analgesia intravenosa.

### Participantes del estudio y criterios de selección

La muestra se obtuvo mediante un muestreo no probabilístico por conveniencia. Se incluyeron inicialmente todos los pacientes adultos atendidos por una SVAE tras un traumatismo registrado en el sistema de información durante el periodo de estudio. Los criterios de inclusión fueron: pacientes mayores de 14 años atendidos con alguno de los códigos CIE relacionados con traumatismos y con criterios clínicos sugestivos de fractura; disponer de registro de escala numérica verbal del dolor al inicio de la asistencia (ENV inicial), con puntuación ≥ 6, y de registro a la llegada al centro hospitalario (ENV final). Los criterios de exclusión fueron: casos sin registro completo de la escala numérica del dolor (ENV inicial y final); casos en los que no se administró analgesia intravenosa; pacientes pediátricos (menores de 14 años); y pacientes sin registro del tiempo de asistencia (diferencia entre la hora de llegada al incidente y la hora de transferencia al hospital de destino). 

### Variables de estudio

Las variables independientes fueron: sociodemográficas (sexo y edad), analgesia administrada, valor inicial de la escala numérica del dolor, código de la unidad asistencial y tiempo de asistencia. Las variables dependientes fueron: valor de la escala numérica verbal del dolor al finalizar la asistencia y número de eventos adversos registrados tras la administración de analgesia. 

### Recolección y análisis de los datos

Los datos se recopilaron del sistema de información SENECA^©^ de la CCUM y las hojas asistenciales del SAMU 061 Baleares, y se trataron de forma anonimizada. El análisis se realizó con el programa *Just Another Statistical Program* (JASP). Las variables cuantitativas se describieron mediante media (), desviación típica (DE) y mediana; las variables categóricas, mediante frecuencias y porcentajes. Antes de realizar las inferencias estadísticas, se evaluó la normalidad de la distribución de las variables cuantitativas mediante la prueba de Shapiro-Wilk. Dado que se constató la falta de normalidad, se emplearon pruebas no paramétricas: prueba de Wilcoxon (W), U de Mann-Whitney (U), prueba de Friedman (χ^2^_F_), coeficiente rho de Spearman (r_s_) y prueba de Kruskal-Wallis (H). Además, cuando se identificaron diferencias significativas, se aplicó la prueba post hoc no paramétrica de Dunn, con correcciones de Holm y Bonferroni. Las asociaciones entre variables se evaluaron mediante pruebas de hipótesis con un nivel de significación p<0.05. Con el fin de reducir el sesgo de reporte, se presentan también los resultados no significativos (p>0.05). Para efectuar las inferencias estadísticas y dada la diversidad de tratamientos administrados, estos se agruparon en cuatro categorías según la familia de principios activos y las combinaciones realizadas durante la asistencia: analgésicos no opioides (paracetamol, metamizol, dexketoprofeno), opioides (cloruro mórfico, fentanilo, meperidina), opioides combinados con analgésicos, opioides combinados con benzodiacepinas (diazepam, midazolam). 

### Aspectos éticos

El presente estudio se ajustó a los principios de la Declaración de Helsinki y contó con la autorización del Comité de Ética de la Investigación Sanitaria de las Islas Baleares (IB5553/24 PI).

## Resultados

El número total de pacientes incluidos en el análisis durante el periodo de estudio se muestra en la Figura 1. 

**Figura 1:** Diagrama de flujo del reclutamiento de la muestra analizada

*SVAE = Soporte vital avanzado de enfermería; ^†^ENV = Escala numérica verbal

En la Tabla 1 se presentan los estadísticos descriptivos de la muestra y las principales variables del estudio.

La edad media de los pacientes fue de 54 ± 21.49 años, y el 48,78% eran mujeres (n=80). El 74,39% (n=122) de las asistencias fueron realizadas por la unidad I210. La puntuación media en la ENV inicial fue de x = 8,48 ± 1.04 puntos, mientras que en la ENV final fue de x = 4,09 ± 2.32 puntos. La reducción media tras la administración del tratamiento fue de x = 4,39 ± 2.39 puntos. En el 5,48% de los pacientes no se observó reducción del dolor percibido (n=9), pese a la administración de analgesia.

**Tabla 1:** Estadísticos descriptivos de las variables analizadas según el recurso de soporte vital avanzado de enfermería (SVAE*), con pruebas de bondad de ajuste (n = 164). Palma de Mallorca, Islas Baleares, España, 2024

Variables por recurso SVAE*	Edad		Tiempo asistencia		ENV^†^ inicial		ENV^†^ final		Diferencia ENV^†^	
	I210^‡^	I212^§^	I210^‡^	I212^§^	I210^‡^	I212^§^	I210^‡^	I212^§^	I210^‡^	I212^§^
Moda	52,85	65,48	42,58	64,27	9,97	8,0	5,42	2,19	3,79	6,05
Mediana	52,0	61,5	41,63	64,34	8,0	8,0	4,0	3,0	4,0	6,0
Desviación típica	20,96	22,26	18,81	22,14	1,41	1,41	2,29	2,20	2,35	2,32
Coeficiente variación	0,40	0,37	0,43	0,34	0,16	0,16	0,52	0,67	0,53	0,44
Rango intercuartílico	33,75	35,25	22,44	23,14	2,0	2,0	3,75	3,0	4,0	3,75
Asimetría	0,08	-0,15	0,81	0,19	-0,33	-0,38	0,03	0,62	0,29	-0,37
Error típico de asimetría	0,21	0,36	0,21	0,36	0,21	0,36	0,21	0,36	0,21	0,36
Curtosis	-0,94	-1,01	1,4	-0,09	-1,17	-0,98	-0,55	-0,28	-0,18	-0,38
Error típico de curtosis	0,43	0,71	0,43	0,71	0,43	0,71	0,43	0,71	0,43	0,71
Shapiro-Wilk	0,97	0,96	0,96	0,98	0,84	0,82	0,96	0,92	0,96	0,95
Valor de p Shapiro-Wilk	0,01	0,22	0,00	0,67	<,001	<,001	0,004	0,008	0,006	0,10
Rango	80,0	80,0	104,6	91,4	4,0	4,0	10,0	8,0	11,0	10,0
Mínimo	15,0	19,0	8,18	21,38	6,0	6,0	0,00	0,00	-1,00	0,00
Máximo	95,0	99,0	112,8	112,8	10,0	10,0	10,0	8,0	10,0	10,0
Primer cuartil	35,25	43,25	30,16	52,32	8,0	8,0	2,25	2,0	2,0	3,25
Tercer cuartil	69,0	78,5	52,6	75,4	10,0	10,0	6,0	5,0	6,0	7,0

*SVAE = Soporte vital avanzado de enfermería; ^†^ENV = Escala Numérica Verbal; ^‡^I210 = Recurso Soporte vital avanzado de enfermería ubicado en la capital Palma de Mallorca; ^§^I212 = Recurso Soporte vital avanzado de enfermería ubicado en la población de Sóller

En la Tabla 2 se presentan las frecuencias de los tratamientos administrados, agrupados por familia de principios activos. 

El análisis bivariado mediante la prueba de rangos con signo de Wilcoxon entre los valores iniciales y finales de la ENV en la muestra total arrojó W=12551, p < 0,001 y un tamaño del efecto r_bis 0,998. Asimismo, el contraste de la U de Mann-Whitney entre la reducción de la ENV en las dos unidades SVAE también mostró un resultado significativo (U= 2720,50, p < 0,005), con un tamaño del efecto negativo (r_bis negativo de -0,258), lo que indica una mayor reducción del dolor en la unidad I212 (Figura 2). 

**Tabla 2:** Frecuencias de la analgesia administrada, agrupada por familias de principios activos (n = 164). Palma de Mallorca, Islas Baleares, España, 2024

Analgesia agrupada	Frecuencia	Porcentaje	Porcentaje válido	Porcentaje acumulado
Analgésicos	25	15,244	15,244	15,244
Opioides	92	56,098	56,098	71,341
Opioide y analgésico	35	21,341	21,341	92,683
Opioide y benzodiacepina	12	7,317	7,317	100,000
Total	164	100,000		

**Figura 2:** Diagrama de caja con las diferencias entre los valores la escala numérica verbal, calculadas como registro inicial menos el final, en los dos recursos de soporte vital avanzado de enfermería analizados

*ENV = Escala Numérica Verbal; ^†^I210 = Recurso soporte vital avanzado de enfermería ubicado en la capital Palma de Mallorca; ^‡^I212 = Recurso soporte vital avanzado de enfermería ubicado en la población de Sóller; ^§^SVAE = Soporte vital avanzado de enfermería

La correlación mediante rho de Spearman entre el tiempo de asistencia (diferencia entre la hora de llegada al incidente y la hora de transferencia al hospital) y la diferencia en la ENV (ENV inicial-ENV final), fue r_s_=0,141, p=0,036, bajo una hipótesis alternativa de correlación positiva. El análisis univariado entre los cuatro grupos de fármacos administrados y la diferencia en la ENV, mediante el contraste de Kruskal-Wallis, indicó diferencias significativas entre los tratamientos agrupados (H(3)= 10,557, p=0,014) y entre los dos recursos implicados (H(1)=7,70, p=0,006), mientras que no sugirió diferencias entre los grupos de tratamiento y el sexo de los pacientes (H(1)=0,456, p=0,499). Las comparaciones *post-hoc* de Dunn, con correcciones de Bonferroni y Holm, mostraron diferencias significativas entre los analgésicos no opioides y los opioides; los resultados se presentan en la Tabla 3. La prueba de Friedman para mediciones repetidas entre los valores inicial y final de la ENV mostró χ^2^_F_ (1)= 150,101, p<0,001. Durante el periodo de estudio no se registraron eventos adversos inmediatos tras la administración de analgesia intravenosa.

**Tabla 3:** Comparaciones *post-hoc* de Dunn entre los diferentes grupos de analgesias administradas (n = 164). Palma de Mallorca, Islas Baleares, España, 2024

Comparación	Z*	W_i_^†^	W_j_^‡^	r_rb_^§^	P^||^	p_Bonf_^¶^	p_Holm_**
Analgésico-opioide	-2,850	55,340	85,592	0,370	0,004	0,026	0,026
Analgésico-opioide+ analgésico	-2,695	55,340	88,557	0,406	0,007	0,042	0,035
Analgésico-opioide+ benzodiacepina	-2,564	55,340	97,708	0,510	0,010	0,062	0,041
Opioide-opioide+ analgésico	-0,317	85,592	88,557	0,034	0,751	1,000	1,000
Opioide-opioide+ benzodiacepina	-0,839	85,592	97,708	0,154	0,402	1,000	1,000
Opioide+analgésico-opioide+benzodiacepina	-0,581	88,557	97,708	0,100	0,561	1,000	1,000

*Z = Valor z en la prueba de Dunn; ^†^W_i_ = Rango promedio del primer grupo que se compara; ^‡^W_j_= Rango promedio del segundo grupo comparado; ^§^r_rb_ = Correlación de rango biserial basada en contrastes de Mann-Whitney individuales; ^||^P = Valor de p comparando z con la distribución normal estándar; ^¶^p_Bonf_ = Valor de p ajustado dividiendo el nivel de significancia entre el número de comparaciones múltiples; **p_Holm_ = Valor de p en la comparación múltiple, ajustado progresivamente al nivel de significancia, empezando con el menor p-valor

## Discusión

La literatura muestra que, en el continente Europeo, la presencia de enfermería liderando determinados modelos asistenciales en las emergencias prehospitalarias está cada vez más extendida; en países como Bélgica, Finlandia, Suecia, Países Bajos, Inglaterra, Gales, Italia o España, para administrar determinados fármacos se requiere que la administración sea realizada por personal de enfermaría acreditado/registrado^11^ ^-^ ^13^. En Italia, algunos estudios describen que la enfermería, con asesoramiento médico o mediante protocolos establecidos, administraba analgesia en unidades de este tipo^14^. En España se está incrementando la implementación de este modelo de recursos SVAE, con variaciones operativas en función de la comunidad autónoma. 

En la CCUM del SAMU 061 Baleares, en 2023, se atendieron 11.916 casos de traumatismos que requirieron la intervención de unidades móviles asistenciales. De estos, 8.667 pacientes fueron trasladados a urgencias hospitalarias; según la literatura, aproximadamente el 70% podría haber padecido dolor^1^ ^-^ ^5^. Estos datos sugieren que aproximadamente 6.066 pacientes podrían haberse beneficiado de tratamiento analgésico, tratamiento que puede ser administrado por las unidades SVAE de forma eficaz y segura.

La valoración y el tratamiento del dolor postraumático llevan años siendo objeto de estudio^15^ y, en la actualidad, continúan siendo una de las piedras angulares del abordaje del paciente traumatizado^16^ ^-^ ^18^; el manejo inadecuado del dolor puede retrasar el retorno al trabajo, reducir la calidad de vida e incrementar el riesgo de complicaciones^19^. En una revisión sistemática sobre la efectividad de las intervenciones realizadas por personal de enfermería en los servicios de urgencias^9^, se identificó como una de las intervenciones más eficaces tanto para reducir el tiempo de espera hasta la administración de analgesia como para disminuir el dolor percibido por los pacientes. Estos hallazgos también se apoyan en los resultados de otro estudio reciente^20^, en el que las enfermeras administraban analgesia temprana a pacientes con probables fracturas de muñeca. 

En la actualidad, incluso se está estudiando y practicando la realización de bloqueos nerviosos ecoguiados a pacientes con fracturas de cadera por personal de enfermería entrenado^21^, intervención contemplada asimismo en las guías más recientes de analgesia para estos pacientes traumatizados^1^ ^,^ ^17^ ^,^ ^22^. Debe destacarse la posibilidad de utilizar la vía intranasal para la administración de fentanilo como una ruta rápida y efectiva, la cual demostró en un estudio que su uso en pacientes con fractura de cadera, en el ámbito de urgencias hospitalarias, fue rápido, seguro y eficaz^23^. 

En la búsqueda bibliográfica realizada se observó una escasez de estudios que evaluaran la administración de analgesia por parte de unidades SVAE. En un estudio italiano^14^ que evaluó la protocolización de la administración de analgesia en el medio extrahospitalario, se describieron amplias diferencias entre las provincias analizadas y un número potencial de pacientes con dolor que no recibían tratamiento adecuado, en relación con la variabilidad en la protocolización de estos procedimientos. Los análisis estadísticos de nuestro estudio evidenciaron una diferencia significativa entre los valores iniciales y finales de la ENV en el conjunto de la muestra (8,48±1,04 DE vs 4,09±2,3 DE , W=12551,0, p<0,001).

Estos resultados pueden compararse con los de otro estudio italiano^5^, en el que, en unidades SVAE que administraban analgesia bajo prescripción médica desde la CCUM, se observó una mejoría significativa entre los valores iniciales y finales (8,36 ± 0,9 vs 4,18 ± 2,2, t-test=6,23, p<0,001, n=25). En nuestro estudio, la reducción media fue superior, = 4,39 ± 2,39 puntos. Por otro lado, en el trabajo italiano^5^ ^)^ las SVAE indicaron haber utilizado cuatro fármacos (paracetamol, ketorolaco, fentanilo y morfina), pero no realizaron análisis comparativo del alivio del dolor producido por cada fármaco ni por sus combinaciones. 

En nuestros datos se observó una mayor reducción de la ENV en la unidad I212 en comparación con la I210. Este hallazgo podría relacionarse con el hecho de que I212 se ubica a aproximadamente 20 km de la capital (Palma de Mallorca), donde se encuentran la mayoría de los hospitales receptores, y con un mayor tiempo de asistencia y traslado (I210 = 42,8 ± 18,81 min vs I212 = 64,38 ± 22,14 min), lo que podría haber permitido más tiempo para alcanzar el efecto analgésico deseado. Por ello, se planteó la hipótesis de que el incremento del tiempo de asistencia y traslado pudiera correlacionarse positivamente con un mayor diferencial entre la ENV inicial y final, al permitir un mayor tiempo para que la analgesia alcance su efecto, especialmente en el caso de analgésicos no opioides, cuyo inicio de acción puede ser más lento. Esta hipótesis se apoyó en la correlación de Spearman, que mostró una asociación baja pero significativa (r_s_=0,141, p<0,05). 

Los opioides son los fármacos más utilizados para el tratamiento del dolor agudo musculoesquelético intenso^24^ ^-^ ^25^. En nuestro estudio, la administración de opioides de forma aislada o en combinación con otros fármacos se asoció con una mayor reducción del dolor percibido (H(3)= 10,557, p=0,014), hallazgo consistente con una revisión sistemática reciente sobre la eficacia de estos tratamientos en el ámbito prehospitalario^26^. No obstante, dicho trabajo señala limitaciones frecuentes en los estudios incluidos, como la ausencia de grupos control, lo que reduce su calidad metodológica; esta limitación también es aplicable a nuestro estudio. 

Por otro lado, se observa una tendencia creciente al uso de estrategias multimodales en el tratamiento analgésico de pacientes traumatizados hospitalizados, con el objetivo de reducir la dosis de opioides y, consecuentemente, sus efectos adversos, sin disminuir el alivio del dolor^27^. Dado que nuestro estudio se centró en pacientes con dolor moderado o intenso según la ENV (> 6 puntos), varias guías clínicas internacionales recomiendan la administración intravenosa combinada de analgésicos no opioides y opioides como tratamiento de primera línea en estos pacientes^17^ ^,^ ^22^, combinaciones que se administraron de forma frecuente en nuestra cohorte.

Entre las limitaciones del estudio se encuentra la posibilidad de haber incurrido en un sesgo derivado de la agrupación de los diferentes grupos de fármacos administrados, puesto que las categorías son amplias y reúnen mecanismos de acción distintos. No obstante, las guías clínicas señalan que, en el manejo del dolor intenso, la clave parece residir en una estrategia analgésica multimodal, que incluye la combinación de fármacos con diferentes mecanismos de acción^17^ ^,^ ^22^ ^,^ ^25^ ^,^ ^28^. Para evaluar este efecto, se realizaron comparaciones *post-hoc* de Dunn entre los grupos de tratamiento y la diferencia obtenida en la ENV, con correcciones de Holm y Bonferroni, que evidenciaron superioridad de los grupos que incluían opioides como principio activo frente a los que recibieron únicamente analgésicos no opioides. 

Este hallazgo puede contrastarse con una revisión sistemática y metaanálisis^2^, en la que se observó que la administración de analgésicos no esteroideos producía una reducción del dolor más efectiva a los 60 min de la administración, en comparación con la administración de opioides en pacientes con traumatismos musculoesqueléticos atendidos en servicios de urgencias. En nuestro estudio, el tiempo de asistencia fue de = 48,33’ ± 21,8 min, por lo que no permite evaluar este efecto en las mismas condiciones; sin embargo, la mayor reducción del dolor observada en la I212 podría sugerir un comportamiento compatible con dicha tendencia.

Además, pudo haberse incurrido en un sesgo de confusión al no haberse comprobado el número de dosis y la concentración administradas en cada caso, puesto que algunos pacientes podrían haber recibido más dosis. No obstante, este efecto podría haberse mitigado gracias a la dosificación ajustada al peso aproximado del paciente, pautada por el médico regulador. Debe destacarse, asimismo, el posible sesgo de selección de los participantes, dado que no se aplicó ningún procedimiento de aleatorización debido al número limitado de pacientes disponibles. Por ello, se optó por incluir a todos los pacientes elegibles durante el período del estudio, con el objetivo de aumentar la representatividad de la muestra y mejorar la validez externa, así como maximizar la obtención de información relevante. 

Otra limitación para considerar es el posible sesgo derivado de la subjetividad de la ENV al medir el dolor percibido por los pacientes. Esta escala puede presentar una variabilidad interobservador significativa^29^. Asimismo, esta variabilidad puede verse influida por sesgos de comprensión y por diferencias culturales asociados a la etnia y/o a las creencias de los pacientes según su origen, factores descritos en una revisión reciente^30^.

Cabe señalar que, a pesar de estas limitaciones, la ENV sigue siendo recomendada por la comunidad científica, junto con la escala visual analógica^1^, como herramienta imprescindible para la valoración del dolor en pacientes adultos por su rapidez y simplicidad^31^ ^-^ ^32^. La utilización de escalas multidimensionales, como la *McGill Pain Questionnaire* (MPQ) o el *Brief Pain Inventory* (BPI), podría atenuar esta limitación^19^; sin embargo, requieren más tiempo para su aplicación. La variabilidad del intervalo entre ambas mediciones de la ENV, al no disponer del registro del momento exacto de cada valoración, pudo producir un sesgo de medición del tiempo. En este estudio se consideró como “valoración inicial” la hora registrada de llegada de la SVAE a la escena y como “valoración final” la hora de transferencia al hospital, lo que aproxima el momento de ambas mediciones. Por otro lado, la ausencia de eventos adversos inmediatos en los pacientes atendidos y medicados por el personal de enfermería de las dos unidades de SVAE aporta evidencia de seguridad a corto plazo. No obstante, este hallazgo solo permite descartar eventos adversos de aparición temprana como reacciones anafilácticas, que suelen presentarse en los primeros 30 min^33^, o episodios de depresión respiratoria tras analgesia y/o sedación, detectables de forma temprana gracias a la monitorización continua, incluida la capnografía^34^, disponible en los monitores de las SVAE. Los eventos adversos tardíos, registrados en la historia clínica hospitalaria, no se consideraron, ya que su evaluación excedía los objetivos específicos del presente trabajo.

## Conclusiones

La administración de opioides a pacientes con traumatismo y dolor moderado o intenso por personal de enfermería en el ámbito extrahospitalario con prescripción médica mediante telemedicina, produce una reducción significativa del dolor según la escala numérica verbal (ENV). La administración de medicación por el personal de enfermería en unidades de soporte vital avanzado de enfermería del SAMU 061, además de efectiva, parece segura, al no registrase eventos adversos a corto plazo. La prolongación del intervalo desde el inicio de la asistencia hasta la llegada al hospital receptor se asocia con una mayor reducción del dolor percibido en estos pacientes.

## Data Availability

Todos los datos generados o analizados durante este estudio están incluidos en este artículo publicado.
